# An Exploration of Socio-demographic, Economic, and Environmental Factors in Black/White Disparities in Low Birth Weight Outcomes: A Cross-Sectional Study

**DOI:** 10.34172/jrhs.2021.49

**Published:** 2021-05-12

**Authors:** Shondra Loggins Clay, Markisha J. Woodson, Renique Kersh

**Affiliations:** ^1^College of Health and Human Sciences, School of Interdisciplinary Health Professions, Northern Illinois University, DeKalb, Illinois, USA; ^2^Department of Public Health, College of Education and Health Services, Benedictine University, Lisle, Illinois, USA; ^3^Office of the President, Vice President for Student Affairs, Simmons University, Boston, Massachusetts, USA

**Keywords:** Healthcare disparities, Vulnerable populations, Pregnancy outcomes

## Abstract

**Background:** Numerous studies have been conducted to seek a better understanding of disparities in adverse pregnancy outcomes. The present study aimed to explore racial differences in influential socio-demographic, economic, and environmental factors in women who have had a low birth weight (LBW) infant (outcome variable).

**Study Design:** A cross-sectional study.

**Methods:** This study used data from the Fragile Families and Child Wellbeing Study (FFCWS). Univariate and multivariate analyses were performed.

**Results:** The obtained results pointed to statistical racial differences between Non-Hispanic (NH) Black and NH White women in the socio-demographic variable of marital status (*P*<0.001). Regarding the assessed economic stability variables, employment status (*P*=0.032), poverty level (*P*<0.001), earnings (*P*=0.038), and federal government assistance paying for rent (*P*=0.007) were statistically significant across the two racial groups. The environmental factors that were statistically significant across racial groups were living in public housing projects (*P*=0.018), car ownership (*P*<0.001), and neighborhood safety (*P*=0.010). The results of the multivariate models revealed that NH Black race and government assistance to pay rent were associated with an increased likelihood of LBW, while being married, having health care coverage, and living in public housing were associated with a decreased likelihood.

**Conclusion:** As evidenced by the obtained results, there were statistically significant racial differences in sociodemographic, economic, and environmental/physical characteristics associated with adverse pregnancy outcomes.

## Introduction

Infant mortality rate (IMR) is recognized as one of the most important indicators of population health, and low birth weight (LBW) has been strongly associated with infant mortality. The United States does not fare well in the area of pregnancy outcomes, as compared to countries with similar sizes and economic profiles. Although the United States has experienced a decrease in IMRs over the years, it has been at a slow rate^1^. Moreover, there are significant racial disparities between Black and White women in the United States. Although there has been an overall decrease in IMRs, the large gap between Black and White women persists. In 2016, IMRs for Black women were more than twice as high as those reported for White women. Black women experienced 11.4 infant deaths per 1,000 live births, while this number was reported as 4.9 for White women, resulting in a 2.3-fold difference in this regard ^2^.

Similar disparities between Black and White women also exist for LBW rates. In 2019, the LBW rate for Whites was 6.9, in comparison to 14.2 for Blacks ^3^. The reduction of racial disparity in the IMR has been identified as a key objective according to Healthy People (HP) 2010, HP 2020, and HP 2030. Healthy People 2030 set the goal at below 5.0 infant deaths per 1,000 live births for all racial groups ^4^. Similar to the previous goals set for HP, it does not appear that the Black population will meet the targeted objective. This consistent disparity requires further analysis and understanding of the factors contributing to adverse pregnancy outcomes, particularly LBW.

Numerous studies have been conducted to seek a better understanding of the disparities in adverse pregnancy outcomes related to racial differences in IMRs and LBW rates between non-Hispanic (NH) Black and NH White women. There is a wealth of literature focused on protective factors and risk factors, such as maternal age, educational attainment, socioeconomic status, marital status, access to care, and maternal health behaviors. A growing literature has consistently pointed to an association between the protective factors and risk factors for pregnancy outcomes, including infant mortality and LBW ^5,6^. Nevertheless, the complexity is exacerbated when considering racial disparities. For instance, related research has posited an association between educational attainment levels and pregnancy outcomes.

Multiple studies have demonstrated that lower educational attainment is closely linked to poor pregnancy outcomes ^4,7^. However, this association is not the same in different groups, and NH Black women may experience worse pregnancy outcomes, regardless of traditional protective factors ^8^. The same disparate outcome across racial group dynamics exists for other protective and risk factors as well. The conundrum is very complex, and there is a need to identify racial differences in contributing factors to adverse pregnancy outcomes and potential barriers to specific group dynamics to assess how to address this issue.

 In light of the aforementioned issues, the present study aimed to explore racial differences in influential sociodemographic, economic, and environmental factors in women with a LBW infant (outcome variable). The guiding research questions were as follows: (1) Are there racial differences between NH Black and NH White women in selected sociodemographic variables and social determinants based on economic stability and social determinants related to neighborhood/physical environment that may explain racial disparities in pregnancy outcomes (specifically LBW outcomes)? (2) Are there sociodemographic variables and social determinants based on economic stability, or environmental variables that are associated with LBW outcomes? The hypotheses assert that there are racial differences in selected variables, and there are factors associated with LBW outcomes.

## Methods

###  Setting and Study Design

The present study was conducted based on a cross-sectional design and made use of data from the Fragile Families and Child Wellbeing Study (FFCWS), which is a cohort study of nearly 5,000 children born to "fragile families" between 1998 and 2000 ^9^. The term “fragile” was used to refer to the participants of the study since most of them were unmarried, had a greater risk of breaking up, and were more likely to live in poverty, as compared to families in which the parents are married and live above the poverty line. The catchment area for the study design included 20 cities in the U.S. with populations larger than 200,000 individuals. The present study focused specifically on socioeconomic indicators, variables related to economic stability, and neighborhood characteristics.

###  Selection of participants: Maternal Race 

In the original database, the maternal race was operationalized by six nominal racial groups and national origin (e.g. White, Black, Asian, American Indian, Other, and Hispanic). There were categories for missing and excluded data, assessed by such categories as refused, do not know, missing, multiple answers, not asked, skipped, or n/a (which were not used in this study). Similar to other studies only assessing Black/White disparities ^10,11^, the populations of interest in this study were only NH Blacks and Whites.

###  Sociodemographic Characteristics

 The four assessed nominal sociodemographic variables were marital status, education level, access to healthcare during pregnancy, and health status. The age of participants was assessed as a continuous ratio variable. Marital status was recorded and defined as a binary variable defined as married (yes) or unmarried (no). In a similar vein, in the present study, education level was dichotomized and categorized as either (1) high school education or higher and (0) less than high school education. Access to healthcare in the FFCWS was measured by the question: “Did you visit a doctor/other health care professional to check on the pregnancy?” Possible responses were categorized as either (1) yes or (0) no. Health status was recorded from the original FFCWS as good health, which measured if individuals self-reported their health status as good, very good, or great. Poor health was measured as a self-reported response of fair or poor health.

###  Economic Stability

 Economic stability was defined by the variables: employment, hours worked per week, poverty categories, income (e.g. general income, income from earnings, from public Assistance, from family or friends, unemployment/SS/disability), federal government help paying for rent, and residual income. Most of the variables were dichotomized as either yes or no (e.g. employment, income, federal government help pay for rent, and residual). Hours worked per week and earnings were continuous ratio variables. Poverty categories were stratified into two categories: below the poverty line and above the poverty line based on the federal poverty measure in the United States. According to the United States Department of Health and Human Services 2021 Poverty Guidelines, the poverty guideline was $26.500 for a family or household of four persons 12. The “below poverty line” categories were defined as 99% or below the poverty guideline and “above the poverty line” was defined as any percentage above 100% of the poverty guideline.

###  Environmental Factors (Neighborhood and Physical Environment)

 Neighborhood and Physical Environment were defined by the following variables: residence in the public housing project, homeownership, car ownership, neighborhood safety, and the length of your residence in the neighborhood. The dichotomized variables with a response of either yes or no were: residence in public housing projects, homeownership, car ownership, and neighborhood safety. The variable, “the length of your residence in the neighborhood” was a continuous ratio variable.

###  Dependent Variable: Low birth weight

 In the current study, the LBW variable was recoded to reflect either (1) had LBW infants or (0) did not. LBW has universally been defined as an infant weighing 2,500 grams (5.5 pounds) or less.

###  Statistical analysis

 Analytical statistics were performed to describe the participants in the study, including the general population and the stratified sample of NH Black and White women. The descriptive analysis analyzed the differences in the variables assessed in the study, including sociodemographic variables, the socioeconomic determinants of health, and the environmental characteristics. Thereafter, univariate and multivariate statistical analyses were carried out. The univariate statistical analysis (chi-square) and ANOVA analytical techniques assessed statistical differences across racial groups. On the other hand, the multivariate statistical analysis assessed the likelihood of having LBW infants given the various social determinants of health. Finally, a correlation matrix was performed to address potential multicollinearity between the variables in the model. All analyses were performed in SPSS software (version 25).

###  Ethics

 The FFCWS data used to conduct the current study was approved by Princeton University, Institutional Review Board (IRB). Informed consent was obtained from all respondents of the survey. All procedures conducted in the study were in accordance with institutional, national, and international ethical standards and guidelines. Data were de-identified to protect the anonymity of respondents.

## Results

###  Descriptive Characteristics

There were 3,256 participants in the general population of NH White and Blacks (Table 1). In the population of NH Whites and Blacks who had LBW infants (n=387), 81 (20.9%) and 306 (79.1%) cases were NH White and NH Black women, respectively. The sociodemographic variables explored in the present study were as follows: marital status, educational attainment level, access to healthcare during pregnancy, and health status. In the general population, the majority of participants were not married, had high educational attainment levels, had access to healthcare, and self-reported good health. Similar results were yielded for women with LBW infants; however, they were significantly different from the general population. In the population of women who had LBW infants, a higher percentage of women were unmarried (*P*<0.001), had lower educational attainment levels (*P*<0.001), no access to healthcare during pregnancy (*P*<0.001), and poorer health (*P*=0.002). The mean age of participants who had LBW infants was reported as 25.38 years (SD=6.681).

**Table 1:** Descriptive characteristics of participants, FFS baseline data, 1998-2000

**Variables**	**General Population** **n=3256**		**Women with LBW** **n = 387**		***P*** **-value**
	**Number**	**Percent**	**Number**	**Percent**	
Race					0.001
White	923	32.2	81	20.9	
Black	1946	67.9	306	79.1	
Marital status					0.001
Married	724	25.4	49	12.7	
Unmarried	2124	74.6	338	87.3	
Education level ^a^					0.001
Less than high school	802	28.0	143	37.0	
High school graduate or higher	2066	72.0	243	62.8	
Access to healthcare during pregnancy ^a^					0.001
Yes	2804	98.2	362	93.5	
No	50	1.8	23	5.9	
Good Health					0.002
Yes	2690	93.9	347	89.7	
No	176	6.1	40	10.3	
Employment (e.g. expected to work next year) ^a^					0.241
Yes	2449	88.4	335	86.6	
No	321	11.6	38	9.8	
Poverty Categories					0.022
Below Poverty line (0-99)	973	33.9	152	39.3	
Above Poverty line (100 +)	1896	66.1	235	60.7	
Income (last year)					0.228
Yes (income)	2635	92.4	353	91.2	
No (no income)	216	7.6	34	8.8	
Income from earnings					0.003
Yes	2049	71.9	250	64.6	
No	801	28.1	136	35.1	
Income from Public Assistance ^a^					0.028
Yes	1069	37.5	165	42.6	
No	1779	62.5	221	57.1	
Income from family/friends					0.182
Yes	880	30.9	129	33.3	
No	1967	69.1	258	66.7	
Income from Unemployment/SS/Disability ^a^					0.099
Yes	225	9.0	43	11.1	
No	2592	91.0	343	88.6	
Federal government helping to pay for Rent ^a^					0.001
Yes	364	12.7	76	19.6	
No	2493	87.3	310	80.1	
Residual Income (end of month) ^a^					0.001
Yes	2214	86.6	275	71.1	
No	344	13.4	70	18.1	
Neighborhood and Physical Environment					
Live in Public Housing Project ^a^					0.431
Yes	329	11.5	46	11.9	
No	2533	88.5	340	87.9	
Home Ownership ^a^					0.033
Yes (own)	1062	37.2	124	32.0	
No (rent)	1791	62.8	260	67.2	
Car ownership ^a^					0.001
Yes	1311	51.1	128	33.1	
No	1256	48.9	222	57.4	
Neighborhood Safety ^a^					0.016
Yes (safe)	2396	83.9	306	79.1	
No (unsafe)	461	16.1	80	20.7	

a^a^Percentages were not added to 100% since categories, such as, "no answer", "don't know/not sure", and "refuse",were excluded from the table.

###  Racial Differences: Analytical Statistics

The results of the present study (Table 2) yielded a statistical racial difference between NH Black and NH White women in sociodemographic variables, the social determinants based on economic stability, and the social determinants related to neighborhood/physical environment. Regarding the assessed sociodemographic variables, within the population of unmarried women, there was a statistically significant higher percentage of NH Black women who had LBW infants had, as compared to NH White women with LBW infants (*P*<0.001). In terms of economic stability factors, NH White women were more likely to be unemployed in the next year, compared to NH Black women (*P*=0.032).

**Table 2:** Descriptive characteristics of participants by race, FFS baseline data, 1998-2000

**Variables**	**All women in study** **LBW, n=387**		**NH White women** **(LBW), n=181**		**NH Black women** **(LBW), n=306**		***P*** **-value**
	**Number**	**Percent**	**Number**	**Percent**	**Number**	**Percent**	
Race							0.001
White	81	20.9	81	100	n/a	0.0	
Black	306	79.1	0	0.0	306	100	
Marital status							0.001
Married	49	12.7	21	25.9	28	9.2	
Unmarried	338	87.3	60	74.1	278	90.8	
Education level ^a^							0.182
Less than high school	143	37.0	26	32.1	117	38.4	
High school graduate or higher	243	62.8	55	67.9	188	61.6	
Access to healthcare during pregnancy ^a^							0.268
Yes	362	93.5	76	96.2	286	93.5	
No	23	5.9	3	3.8	20	6.5	
Good Health							0.534
Yes	347	89.7	73	90.1	274	89.5	
No	40	10.3	8	9.9	32	10.5	
Economic Stability							
Employment (e.g. expected to work next year) ^a^							0.032
Yes	335	86.6	65	83.3	270	91.5	
No	38	9.8	13	16.7	25	8.5	
Poverty Categories							0.001
Below Poverty line (0-99)	152	39.3	14	17.3	138	45.1	
Above Poverty line (100 +)	235	60.7	67	82.7	168	54.9	
Income (last year)							0.581
Yes (income)	353	91.2	74	91.4	279	91.2	
No (no income)	34	8.8	7	8.6	27	8.8	
Income from earnings							0.038
Yes	250	64.6	59	73.8	191	62.4	
No	136	35.1	21	26.3	115	37.6	
Income from Public Assistance ^a^							0.116
Yes	165	42.6	29	36.3	136	44.4	
No	221	57.1	51	63.7	170	55.6	
Income from family/friends							0.176
Yes	129	33.3	31	38.3	98	32.0	
No	258	66.7	50	61.7	208	68.0	
Income from Unemployment/SS/Disability ^a^							0.553
Yes	43	11.1	9	11.3	34	11.1	
No	343	88.6	71	88.8	272	88.9	
Federal government helping to pay for Rent ^a^							0.007
Yes	76	19.6	8	9.9	68	22.3	
No	310	80.1	73	90.1	237	77.7	
Residual Income (end of month) ^a^							0.313
Yes	275	71.1	57	82.6	218	79.0	
No	70	18.1	12	17.4	58	21.0	
Live in Public Housing Project ^a^							0.018
Yes	46	11.9	4	4.9	42	13.8	
No	340	87.9	77	95.1	263	86.2	
Home Ownership ^a^							0.106
Yes (own)	124	32.0	31	38.8	93	30.6	
No (rent)	260	67.2	49	61.3	211	69.4	
Car ownership ^a^							0.001
Yes	128	33.1	44	62.0	84	30.1	
No	222	57.4	27	38.0	195	69.0	
Neighborhood Safety ^a^							0.010
Yes (safe)	306	79.1	72	88.9	234	76.7	
No (unsafe)	80	20.7	9	11.1	71	23.3	

Percentages were not added to 100% since categories, such as, "no answer", "don't know/not sure", and "refuse", were excluded from the table.

 Nonetheless, NH Black women had a higher percentage of the population who were below the poverty line (P<0.001), had no income (P=0.038), and received federal government assistance to pay for rent (P=0.007). The assessment of the environmental factors indicated that NH Black women who had low infants had higher percentages of women who lived in public housing projects (P=0.018), did not own a car (P<0.001), and lived in unsafe neighborhoods (P=0.010).

A binary logistic regression model (Table 3) was performed to analyze the predictors of having LBW infants based on sociodemographic characteristics, social determinants categorized by economic stability, and social determinants assessed by the measures of the neighborhood and physical environment. Furthermore, a correlation matrix was employed to address potential multicollinearity among the variables in the model, in which the variables were uncorrelated (less than 0.3 correlation reported). NH Blacks were 1.54 times (P=0.010) more likely to have LBW infants, as compared to NH Whites. The only social determinant of health that was statistically significant and had a positive association with an increased likelihood of LBW was receiving assistance from the federal government to pay for rent (OR=1.62, P=0.009).

 Furthermore, being married (OR=.55, P=0.003), having health care coverage (OR=.35, P<0.001), and living in public housing (OR=.64, P=0.031) were associated with a decreased likelihood of having LBW infants. After controlling for race, significant predictors of LBW for NH Blacks were access to health care during pregnancy (OR=.351; P=0.002), federal government assistance to pay for rent (OR=1.674; P=0.009), and living in public housing projects (OR=.610; P=0.022). Being married (P=0.042) and having good health (P=0.047) were both significant predictors that were associated with a decreased likelihood of LBW for NH Whites.

## Discussion

 The impetus of this study was to specifically focus on a vulnerable seemingly homogenous population of women. Data from the Fragile Families and Child Wellbeing Study (FFCWS) was used since respondents were referred to as “fragile”, meaning that most of them were unmarried, had a greater risk of breaking up, and more likely to live in poverty, compared to families in which the parents are married and live above the poverty lines. The results obtained from our general population supported a “fragile” population. As expected, the majority of women were unmarried, had lower educational attainment, and experienced financial challenges. However, statistically significant differences emerged when the results were disaggregated to explore the women with LBW infants and differences in NH White and NH Black women.

The women who had LBW infants had more significant challenges, compared to the general population. Regarding the assessed sociodemographic variables, there was a higher percentage of unmarried women with lower educational attainment levels and no access to health care during pregnancy. Multiple studies have demonstrated that selected sociodemographic variables are associated with poor pregnancy outcomes^4,6,8^, and the results of the current study supported these findings. The individuals who had adverse pregnancy outcomes exhibited higher percentages in risk factors, as compared to those in the general population.

**Table 3:** Logistic Regression Model analyses of participants, FFS baseline data, 1998-2000

**Variables**	**Odds Ratio (95% CI)**	***P*** **-value**
Racial Characteristics		
White	1.00	
Black	1.54 (1.11, 2.122)	0.010
Marital Status		
Unmarried	1.00	
Married	0.55 (0.37,.808)	0.003
Educational Level		
Less than HS	1.00	
HS Education or higher	0.92 (0.70, 1.205)	0.533
Access to healthcare during Pregnancy	
No healthcare coverage	1.00	
Healthcare coverage (any)	0.35 (0.19, 0.628)	0.001
Health Status		
Poor health	1.00	
Good health	0.67 (0.45, 1.007)	0.054
Employment		
Not employed	1.00	
Employed	0.92 (0.61, 1.38)	0.670
Poverty Categories		
Below poverty line	1.00	
Above Poverty Line	1.12 (0.84, 1.50)	0.427
Income (last year)		
Yes (income)	1.00	
No (no Income)	1.45 (0.85, 2.47)	0.169
Income from earnings		
No income from earnings	1.00	
Income from earnings	0.97 (0.71, 1.33)	0.863
Income from Public Assistance		
No public assistance	1.00	
Public Assistance	0.86 (0.64, 1.15)	0.298
Income from family/friends		
No income support family/friends	1.00	
Income support family (ref=no income support family)	1.10 (0.85, 1.43)	0.456
Income from Unemployment/SS/Disability		
No income from unemployment/SS/Disability	1.00	
Income from unemployment/SS/Disability	1.22 (0.83, 1.81)	0.315
Federal Government helping to pay for Rent		
No federal Government Assistance to Pay for rent	1.00	
Federal Government Assistance to Pay for rent	1.62 (1.13, 2.33)	0.009
Residual Income		
No residual income	1.00	
Residual Income	0.78 (0.57, 1.07)	0.124
Live in Public Housing Project		
No public housing	1.00	
Public Housing	0.64 (0.43, 0.96)	0.031
Home ownership		
Do not own home (rent)	1.00	
Own Home	1.05 (0.81, 1.38)	0.704
Own a car		
Do not own car	1.00	
Own Car	0.78 (0.59, 1.03)	0.078
Neighborhood Safety		
Unsafe neighborhood	1.00	
Safe Neighborhood	0.96 (0.71, 1.30)	0.782

Further synthesis of the data by racial group dynamics revealed that there were significant racial differences in women who had LBW infants. There was a statistically significant racial difference between NH Black women and NH White women in the sociodemographic variable of marital status. Robards (2012) ^13^, Joung (1997) ^14^, and Gizaw (2018) ^15^ posited that marital status is a protective factor leading to better health and pregnancy outcomes. The higher percentage of unmarried NH Black women may explain the higher percent of LBW rates within this population.

For the assessed economic stability variables, employment status, poverty level, income, and federal government assistance paying for rent were statistically significant across NH Black/NH White racial groups. NH White women had a higher percentage of individuals who lived above the poverty line, had earnings, and received less assistance from the federal government for rent. On the contrary, NH Black women had higher percentages of individuals who were below the poverty line, had lower income from earnings, and received more assistance from the federal government. In a study exploring socioeconomic inequalities in low birth weight, Martinson and Reichman (2016) observed an explicit association between low income and low birth weight ^16^. Similarly, Komro et al. (2016) explored if there was an association between increased minimum wage and adverse pregnancy outcomes (e.g. infant mortality and birth weight) ^17^. The findings of the mentioned study indicated that increases in the minimum wage resulted in decreases in LBW/postneonatal mortality (approximately 1-4% decrease). The results of the referred study have implications for the present research. In a “fragile” population where women may receive minimum wage income, a slight increase in income and economic mobility can possibly lead to positive pregnancy outcomes. The reduction of the economic income gap between NH Black and NH White women may lead to better pregnancy outcomes for NH Black women.

In contrast to a study which indicated that NH Black women had lower employment levels^10^, in the present research, a higher percentage of NH White women stated that they were not expecting to work next year (e.g. had a lower percentage of employment levels). Yoon & Waite (1994) explained that there are differences in sociodemographic variables and social determinants (e.g. education levels and household income earnings) that may require NH Black women to return to work at faster rates, compared to NH White women^18^. This may possibly explain why higher employment levels were observed for NH Black women; nonetheless, this area warrants further research.

Other factors that were statistically significant include residence in public housing projects, car ownership, and neighborhood safety. A recent study exploring LBW disparities between NH Black and NH White women found several factors associated with an increased likelihood of LBW, including the use of government resources to pay for infants' birth and residence in governmental housing^19^. In a similar study exploring the relationship between racial disparities and low birth weight risk, Clay and Andrade (2016) identified the use of government assistance (e.g. receiving government/public assistance) as factors associated with pregnancies that result in LBW ^14^.

In their study, Yangyuen et al (2020) reported that perceived neighborhood crime exerted an impact on risk behaviors (e.g. alcohol use) that can possibly lead to adverse health outcomes ^20^. In the current study, NH Blacks who had LBW infants were more likely to have such systemic barriers as heavy reliance on the government for assistance, residence in public housing projects, absence of transportation, and residence in unsafe neighborhoods. The lower levels of economic stability and perilous neighborhood/physical environment conditions increase the risk for adverse pregnancy outcomes for NH Black women, compared to their White counterparts. Although the variables may not fully explain the disparity, the results provide evidence for why racial and economic differences are a major concern related to adverse pregnancy outcomes.

 The findings related to an increase or decrease likelihood of LBW indicated most importantly that NH Blacks were 1.54 times more likely to have LBW infants, compared to NH Whites. The results are not different from the current literature. This is more evidence that effective policies and interventions are needed to address this health issue. After controlling for race, the significant predictor associated with an increased likelihood of LBW for NH Blacks was federal government assistance to pay for rent. The factors associated with a decreased likelihood were access to health care during pregnancy and residence in public housing projects.

The increased likelihood of LBW for federal government assistance to pay for rent aligns with the findings reported by Clay and Andrade (2016)^14^. Moreover, it affirms that economic stability for NH Blacks may bridge the gap in disparate outcomes. In a similar vein, it is important to ensure that NH Black women have access to health care during pregnancy. Numerous studies have posited the benefits of access to health care. In a study exploring socioeconomic inequalities in healthcare utilization, Rezaeian (2018) found that health care coverage was associated with increased utilization of inpatient care, and socioeconomic status was a main contributing factor to outpatient care utilization^21^.

The findings highlighted the need for affordability and accessibility of health care, and it may be even more imperative to ensure that NH Black women have this access during pregnancy to reduce LBW rates. It was also observed that living in public housing projects was associated with a decreased likelihood of LBW for NH Blacks. Swope et al. (2019) extensively discussed housing as a social determinant of health, particularly health equity^22^. The authors asserted that housing is a pathway to the reduction of disparities. Aligned with this hypothesis, the findings of the current study may further highlight the importance of having access to any type of housing in the reduction of adverse pregnancy outcomes, specifically for NH Black women.

Being married (P=0.042) and having good health (P=0.047) were both significant predictors associated with a decreased likelihood of LBW for NH Whites. Clay et al. (2016) found that the women who self-reported poor health were 3.7 times more likely to have infants with low birth weight, as compared to their counterparts who self-reported good health ^14^. Therefore, it is no surprise that marital status and good health were associated with a decreased likelihood of LBW. Nonetheless, more extensive research is needed in this area since there were no other significant predictors of LBW for NH Whites. In addition, further research is needed to assess why marital status and good health status serve as predictors of LBW for NH Whites and not for NH Blacks.

 The data set used for the current study included self-reported data through the FFCWS. The interviewed mothers were asked to report on perceptions of their health, leaving the researchers to trust the reported perceptions as an accurate statement of health status. Several years of FFCWS data suggest consistency in reporting outcomes; however, it is important to acknowledge the limitations of self-reported data. The current study primarily focused on the perceptions of mothers; nonetheless, the FFCWS also includes the perceptions of fathers and caregivers, which upon further exploration, may provide deeper insight into factors associated with racial and economic disparities between Black and White pregnant mothers.

 Furthermore, the low r-squared of the logistic regression models was acknowledged; however, our omnibus tests of model coefficients yielded statistical significance (P<0.05). Even though the line of best fit may indicate “not the best model fit” for the data, the trends demonstrated that the predictor variables are still associated with the outcome variable. This could be attributed to variability in the prediction intervals and needs further exploration in the future.

## Conclusion

 Marked differences between NH Black and NH White women with LBW infants, as well as economic situation, housing, and medical characteristics, were significantly associated with adverse pregnancy outcomes. The protective and risk factors for NH Blacks relative to employment, the use of governmental support, and environmental safety were strongly associated with LBW. Although aggregate data confirmed that these factors concern both NH Blacks and Whites, NH Blacks were at a much higher level of risk for adverse pregnancy outcomes when disaggregated by race. The results of the analyzed data pointed to significant racial and economic disparities that explain differences in pregnancy outcomes, and the results have future implications for policies and interventions.

## Acknowledgment

 The authors' deepest appreciation goes to the School of Interdisciplinary Health Professions (SIHP) at Northern Illinois University and the Department of Public Health at Benedictine University for their unwavering support.

## Conflict of interest

 The authors declare that they have no conflict of interest regarding the publication of the current article.

## Funding

 The present research project was supported by the Eunice Kennedy Shriver National Institute of Child Health and Human Development (NICHD) of the National Institutes of Health (R01HD036916, R01HD039135, and R01HD040421), as well as a consortium of private foundations. The content is solely the responsibility of the authors and does not necessarily represent the official views of the National Institutes of Health.

## Highlights

- NH Black women were more likely to have a LBW infant, compared to NH White women.
- NH Black women had more risk factors associated with adverse pregnancy outcomes.
- Race and government assistance were associated with an increased likelihood of LBW.
